# Modified procedure of anterior orbital exenteration enables eye socket reconstruction: A retrospective cohort study

**DOI:** 10.1097/MD.0000000000028698

**Published:** 2022-01-28

**Authors:** Ruiqi Ma, Xiaofeng Li, Lu Gan, Jie Guo, Jiang Qian

**Affiliations:** a Department of Ophthalmology, Fudan Eye & ENT Hospital, Shanghai, China; b Laboratory of Myopia, Chinese Academy of Medical Sciences, Shanghai, China; c NHC Key Laboratory of Myopia, Fudan University, Shanghai, China.

**Keywords:** cosmetic rehabilitation, ocular surface malignancy, orbital exenteration, socket reconstruction

## Abstract

The conventional procedure of anterior orbital exenteration is unfavorable for eye socket reconstruction, whereas a modified procedure enables socket reconstruction and prosthesis fitting. Our study aims to compare the cosmetic outcomes between these 2 surgical techniques.

We retrospectively recruited patients treated with modified or conventional exenteration during January 2015 to May 2021 in our hospital. The conventional approach was performed along with dermis-fat graft transplantation. The modified approach was conducted followed by eye socket reconstruction and eyelid blepharoplasty. The clinical data were collected and analyzed, including demographics, tumor characteristics, postoperative complications, tumor-related events, and cosmetic outcomes.

Forty-nine patients were consecutively recruited in this study, including 22 cases of modified exenteration and 27 cases of conventional exenteration. Forty-four subjects (89.8%) were diagnosed with ocular surface malignancies (conjunctival melanoma and squamous cell carcinoma) and 5 subjects (10.2%) were diagnosed with extraocular stage of uveal melanoma. After follow-up for 31.8 ± 17.1 months, the 1-, 2-, 5-year overall survival rate was calculated as 100%, 79.2%, and 59.2% in the Modified group, and 94.2%, 73.8%, and 51.5% in the Conventional group. Comparison of the survival curves showed no significant differences. In the Modified group, all patients received orbital implant placement and eye socket reconstruction. The implant motility was satisfactory in 12 cases (54.5%) with movements in 3 to 4 directions. The eyelid function was acceptable in 17 cases (77.3%) with no entropion, ectropion or lower lid laxity. Ocular prosthesis was delivered in 17 cases (77.3%) with successful fitting in 11 cases (64.7%). The self-rated cosmetic score was statistically (*t* test, *P* < .0001) higher in the Modified group (6.7 ± 0.9) than the Conventional group (2.2 ± 0.4).

The modified approach to anterior orbital exenteration enables eye socket reconstruction and cosmetic rehabilitation while still preserves the curable chance for the treatment of advanced periocular/intraocular malignancies.

## 1 Introduction

**Figure 1:**
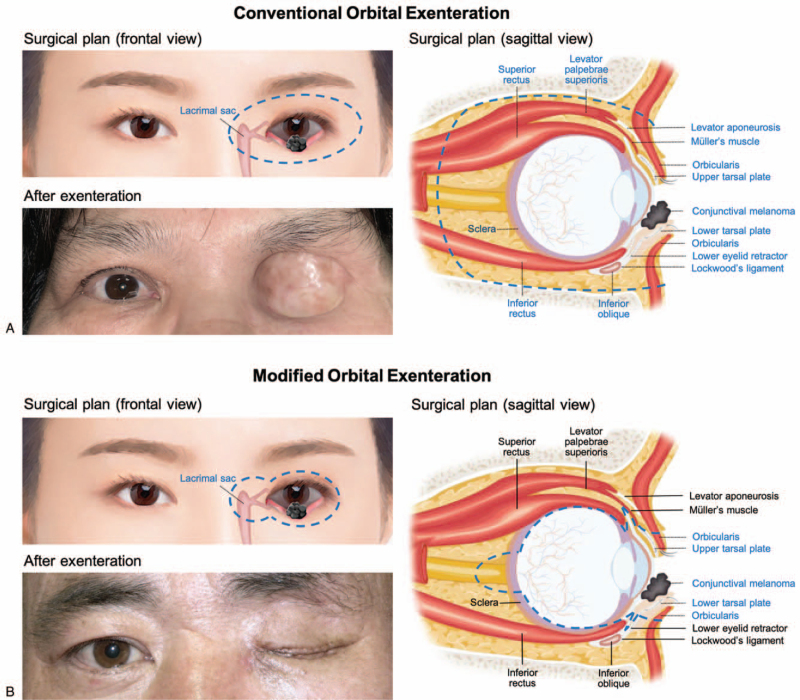
Surgical plan and cosmetic outcome. (A) Conventional orbital exenteration. (B) Modified orbital exenteration. The surgical areas are circled in dashed blue lines. The removed structures are illustrated with dotted lines and innovations in blue. The retained structures are illustrated with solid lines and innovations in black. The typical cosmetic outcomes are presented in frontal view.

Anterior orbital exenteration is indicated for the treatment of advanced periocular or intraocular tumors, among which squamous cell carcinoma (SCC), conjunctival melanoma (CM), and the extraocular stage of uveal melanoma are the most frequent tumor types.^[1–2]^ The conventional technique requires removal of the eyelids, the globe, the lacrimal sac as well as the frontier part of extraocular muscles, and orbital fat, resulting into a hollow appearance and little chance of eye socket reconstruction (Fig. 1A). The disfiguring effects and prolonged rehabilitation time seriously deteriorate patients’ quality of life. Many efforts have been made to optimize the surgical plan of exenteration so as to minimize the deforming effect as well as maximize the curable chance.^[3]^ The modified techniques include eyelid sparing approach, globe sparing approach, skin–muscle sparing approach, and the most ideally individualized approach.^[4–6]^ Several factors should be taken into account to make individualized surgical decisions, for instance, the biology of the tumor, the anatomic location, the potential tissue planes to achieve adequate margins, and the patient's goal for cosmetics.^[7]^ Our previous study retrospectively reviewed the medical records of advanced CM cases treated with individualized exenteration and compared the long-term outcome between the individualized and conventional techniques.^[8]^ The follow-up data suggested that the individualized approach offers improved aesthetic results while still maximizes the survival rate for advanced CM. According to our experience, we standardized and simplified the procedure of minimally invasive exenteration which enables eye socket reconstruction and ocular prosthesis fitting (Fig. 1B). This report aims to describe the operative details of modified exenteration, compare the rehabilitative results between the conventional and modified techniques, and discuss about the strengths and weaknesses of the modified exenteration approach.

## 2 Patients and methods

### 2.1 Patient recruitment

Patients who underwent conventional (the Conventional group) or modified (the Modified group) surgery of anterior exenteration in our hospital from January 2015 to May 2021 were recruited in this study. The surgical indications were advanced periocular or intraocular malignancies, which originated from the ocular surface (conjunctive and cornea) or the anterior segment of globe (iris and ciliary body) and invaded the adjacent structures including the superficial sclera of globe, the tarsal part of eyelid skin, and the anterior one third of orbit. The contraindications for modified exenteration were imaging-confirmed or macroscopic tumor invasion of lacrimal sac, nasolacrimal duct, and paranasal sinuses. Notably, local invasion of nasolacrimal system and paranasal sinuses was not contraindication for the conventional exenteration technique. Moreover, both procedures were performed as palliative treatment for patients with regional lymph node metastasis and distant metastasis. The medical records were collected during May 2021 to October 2021, including demographics, tumor characteristics, postoperative complications, rehabilitation, cosmetic results, and survival rate. The study protocol was in accordance with the Declaration of Helsinki and was approved by the Ethics Committee of our hospital. Informed consents were obtained from all patients.

### 2.2 Surgical procedure

In the Conventional group, the exenteration surgery was performed along with dermis-fat graft transplantation for orbital reconstruction (Fig. 1A). In the Modified group, the total procedure requires 3 stages of surgery, including modified exenteration (see Supplemental Digital Content 1, video which demonstrates the procedure of modified orbital exenteration), socket reconstruction (see Supplemental Digital Content 2, video which demonstrates the procedure of eye socket reconstruction), and eyelid blepharoplasty.

**Figure 2:**
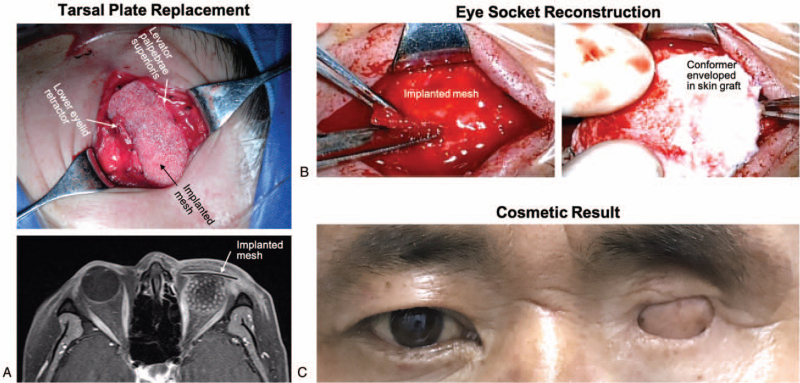
Surgical techniques for eye socket reconstruction. (A) The levator palpebrae superioris and lower eyelid retractor were sutured to a biocompatible mesh which replaced the tarsal plates. The mesh was clearly viewed on enhanced T1-weighted MRI scan. (B) The implanted mesh was cut into 2 pieces, and a conformer-enveloped dermal graft was sutured underneath to reconstruct the eye socket. (C) The final cosmetic result of eye socket reconstruction is presented.

The operative details of the modified approach is described as below. After general anesthesia, methylene blue (a nontoxic dying reagent for epithelial tissues) was applied to the conjunctival sac to mark the ocular surface area. After subcutaneous infiltration of lidocaine and adrenaline to enhance hemostasis, a cutaneous incision was made with a 3 mm safe margin away from the tumor-infiltrated eyelid skin (Fig. 1B). Dissection was carried out through the orbicularis muscle until reaching the orbital septum. Then the skin of upper and lower eyelids were sutured together so as to avoid tumor cell dissemination during the following procedures. The levator palpebrae superioris and lower eyelid retractor, which are important for eyelid reconstruction, were freed and marked with 5-0 silk suture. Further dissection was carried out to reach the sclera, and the sclera was incised with a 8 to 10 mm margin away from the blue-stained conjunctival sac. A standard evisceration procedure was performed, and an orbital implant was placed into the scleral shell to achieve adequate volume replacement. The lacrimal sac was separated with periosteal elevator, and the nasolacrimal duct was transected with monopolar cautery. At the end of the surgery, the levator palpebrae superioris and lower eyelid retractor, which were marked with 5-0 silk suture, were sutured to a biocompatible mesh (Fig. 2A). The mesh could be customized to replace tarsal plates during the second stage of eye socket reconstruction. The skin wound was closed with 5-0 silk suture, and a compressive dressing was then applied for 7 days to provide hemostasis.

After the first surgery, the tumor was removed and processed for pathologic analysis. Patients with positive tumor margins were further treated with adjuvant radiotherapy or chemo-radiotherapy according to the pathologic results. The follow-up plan was every 3 months during the first year and every 6 months during the second year. After 2-year follow-up, cases without local recurrence underwent eye socket reconstruction. After general anesthesia and subcutaneous infiltration of lidocaine and adrenaline, skin incision was made at the proposed site of palpebral fissure. After dissection of the levator palpebrae superioris and the lower eyelid retractor along the implanted mesh, the mesh was cut into 2 pieces and tailored to the shape of tarsal plates to provide mechanical support (Fig. 2B, left). A dermal graft harvested from the subclavian region was sutured into an envelope shape with the dermis layer flipped outside. A convex–concave conformer was wrapped inside to maintain the eye socket shape and restore the socket volume (Fig. 2B, right). The conformer-enveloped dermal graft was then sutured beneath the implanted mesh, and the eyelid skin was sutured to both the mesh and the dermal graft to reconstruct functional eyelids. After suture together the palpebral fissure, a compressive dressing was applied for 10 days to allow the dermal graft secured to the surrounding tissues. The final stage of surgery was performed 3 months later with a simple procedure of palpebral fissure incision for eyelid blepharoplasty. One month later, the patients achieved the final cosmetic results (Fig. 2C).

### 2.3 Cosmetic assessment

The assessment of eye socket was performed in the Modified group according to the following items. The implant motility was assessed based on movement directions. The eyelid function was evaluated in terms of entropion, ectropion, and lower eyelid laxity. The inferior fornix depth was measured using a ruler after eye socket reconstruction. The degree of eye socket contracture was graded according to Krishna classification^[9]^ as follows: Grade 1, shallow or shelved lower fornix; Grade 2, loss of both upper and lower fornices, preventing retention of artificial eye; Grade 3, included loss of all 4 fornices; Grade 4, loss of all fornices along with reduction of palpebral aperture; Grade 5, severely contracted socket with recurrence of contracture following repeated trials of reconstruction. Patients wearing prosthesis were rated for the degree of prosthesis fitting.^[10]^ A fitting was deemed to be successful when the prosthesis fit appropriately between the superior and inferior fornices without rotation or slipping. A fitting was deemed to be acceptable if the prosthesis fitted without extrusion but may tilt or rotate slightly while blinking. A fitting was deemed to be poor if there was a shallow fornix, with a depth that was inadequate to maintain the prosthesis. The patients’ satisfaction of cosmetic results was classified as satisfied, acceptable and unsatisfied based on the results of a questionnaire,^[10]^ and the results were compared between the Modified and Conventional groups.

### 2.4 Statistical analysis

Data were analyzed with Statistical Product and Service Solutions (V10.10). Student unpaired *t* test was used for continuous variables. Two-tailed χ^2^ test or Fisher exact test was used for categorical variables as appropriate. Wilcoxon rank-sum test was used for ordinal variables. Cumulative survival rates were calculated by Kaplan–Meier curves and compared by log-rank test. A value of *P* < .05 was considered statistically significant.

## 3 Results

Forty-nine patients were consecutively recruited in this study, including 22 cases in the Modified group and 27 cases in the Conventional group (Table 1). Follow-up data were available for all patients with a mean follow-up duration of 31.8 ± 17.1 months. In the Modified group, positive surgical margin was detected in 3 cases (13.6%), 2 of which were reported as microscopic tumor residue in the nasolacrimal system. The overall survival rate was 100% at 1 year, 79.2% at 2 years, and 59.2% at 5 years; the metastasis-free survival rate was 94.4% at 1 year and 74.4% at 2 years. In the Conventional group, the tumor node metastasis (TNM) stage was slightly but not significantly more advanced than the Modified group (Table 1). The overall survival rate was 94.2% at 1 year, 73.8% at 2 years, and 51.5% at 5 years; the metastasis-free survival rate was 90.1% at 1 year and 63.7% at 2 years. Comparison of the survival curves between the 2 study groups revealed no significant differences.

**Table 1:** Clinical characteristics of recruited subjects.

Items^∗^	Modified (*N* = 22)	Conventional (*N* = 27)	*P* value
Age (yr)	60.7 ± 11.8	65.2 ± 14.5	.247
Sex			.372
Male	12 (54.5)	19 (70.4)	
Female	10 (45.5)	8 (29.6)	
Laterality			.779
Left	10 (45.5)	11 (40.7)	
Right	12 (54.5)	16 (59.3)	
Tumor			.973
CM	15 (68.2)	18 (66.7)	
SCC	5 (22.7)	6 (22.2)	
UM (extraocular stage)	2 (9.1)	3 (11.1)	
TNM staging^†^			
T category			.597
T3a	5 (22.7)	5 (18.5)	
T3b	12 (54.5)	13 (48.1)	
T3c	5 (22.7)	7 (25.9)	
T3d	0	2 (7.4)	
N category			.715
N0	19 (86.4)	22 (81.5)	
N1	3 (13.6)	5 (18.5)	
M category			NA
M0	21 (95.5)	26 (96.3)	
M1	1 (4.5)	1 (3.7)	
After orbital exenteration			
Surgical margin			.715
Negative	19 (86.4)	22 (81.5)	
Positive	3 (13.6)	5 (18.5)	
Adjuvant treatment			.951
Radiotherapy	1 (4.5)	3 (11.1)	
Chemotherapy	2 (9.1)	4 (14.8)	
Chemo-radiotherapy	3 (13.6)	6 (22.2)	
Follow-up duration (mo), mean ± SD	28.9 ± 16.9	34.2 ± 17.3	.287
Follow-up results			.468
Tumor-free survival	15 (68.2)	14 (51.9)	
Tumor-bearing survival	2 (9.1)	5 (18.5)	
Tumor-related death	5 (22.7)	8 (29.6)	

CM = conjunctival melanoma, NA = not applicable, SCC = squamous cell carcinoma, SD = standard deviation, UM = uveal melanoma.

∗ The continuous variables are expressed as mean ± standard deviation, and the categorical variables are reported as counts (percentage).

† The TNM stage is categorized according to the clinical scale of *Cancer Staging Manual* (eighth edition) published by the American Joint Committee on Cancer. The T stage indicates local invasion of globe (T3a), eyelid (T3b), orbit (T3c) and lacrimal sac/nasolacrimal duct/paranasal sinuses (T3d). The N stage indicates no evidence of lymph node involvement (N0) or metastasis in regional lymph nodes (N1). The M stage indicates no metastasis (M0) or distant metastasis (M1).

In the Modified group, no infection or postoperative fistula were reported after orbital exenteration, and 1 case experienced hematoma due to early removal of compressive dressing (Table 2). The complications were mostly associated with radiotherapy, including 1 case of dermatitis and 2 cases of delayed wound healing (Table 2). In the Conventional group, postexenteration complications included 2 cases of socket infection, 1 case of ethmoidal fistula, 3 cases of wound delay, and 4 cases of dermatitis. The incidence of complication was slightly higher in the Conventional group (37.0%) than the Modified group (18.2%).

**Table 2:** Reconstructive outcomes of the subjects underwent modified exenteration.

Items^∗^	Results	Items	Results
Postoperative complications		Cosmetic Results	
Socket infection	0	Inferior fornix depth (mm)	10.7 ± 8.0
Ethmoidal fistula	0	Socket contracture	
Hematoma	1 (4.5)	Grade 1	3 (13.6)
Need of reoperation	0	Grade 2	1 (4.5)
Wound healing delay	1 (4.5)	Grade 3	0
Dermatitis	2 (9.1)	Grade 4	0
Functional results		Grade 5	0
Implant motility		Prosthesis fitting	
Motility in 4 directions	5 (22.7)	Successful fitting	11 (50.0)
Motility in 3 directions	7 (31.8)	Acceptable fitting	5 (22.7)
Motility in 2 directions	5 (22.7)	Poor fitting	1 (4.5)
Motility in 1 direction	3 (13.6)	No prosthesis delivered	5 (22.7)
No motility	2 (9.1)	Patients’ satisfaction	
Entropion	4 (18.2)	Satisfied	7 (31.8)
Ectropion	0	Acceptable	12 (54.5)
Lower lid laxity	1 (4.5)	Unsatisfied	3 (13.6)

∗ The continuous variables are expressed as mean ± standard deviation (range), and the categorical variables are reported as counts (percentage).

In the Modified group, all patients received orbital implant placement, and the implant motility was satisfactory in 12 cases (54.5%) with movements in 3 to 4 directions (Table 2). Three patients (13.6%) complaint of hollow socket, while the others considered acceptable or satisfied with their appearance. Seventeen patients (77.3%) achieved good eyelid function without entropion, ectropion or lower lid laxity. Four patients (18.2%) suffered eye socket contracture with an average inferior fornix depth calculated as 5.9 ± 2.4 mm. Seventeen patients received prosthesis delivery with successful fitting in 11 cases (64.7%). Eye socket reconstruction was not performed in the Conventional group, and 22 patients (81.5%) were unsatisfied with the cosmetic results. The cosmetic score was rated as 2.2 ± 0.4 on a scale of 1 to 10 in the Conventional group during the last follow-up, which was statistically lower than an average of 6.7 ± 0.9 (*t* test, *P* < .0001) in the Modified group. Notably, after browsing the photos of conventional exenteration, patients in the Modified group raised the cosmetic score to 9.3 ± 0.6 (*t* test, *P* = .0001), suggesting a significant improvement of the patients’ satisfaction.

## 4 Discussion

Compared with the previous methods, the exenteration technique reported in our study shows unique strengths. First, this technique provides a simplified, standardized procedure to tailor the extent of resected tissues, making it easy for specialists to learn. Second, this technique enables to maximize the preserved tissues, allowing rapid healing and early referral if adjunctive orbital radiotherapy is needed. Third, this technique requires no extra volume replacement except orbital implant, which shortens the operation time and avoids hollow appearance during long-term follow-up. Last but not least, even though this procedure requires 3 stages of surgery, patients could benefit from the second and third operations. In previous studies, patients who underwent orbital exenteration should wear adhesive-retained or spectacle-retained prosthesis to camouflage ocular surface structures. In our study, however, the palpebral fissure and eyelids were reconstructed, enabling patients to wear removable ocular prosthesis instead of large sticky pad. Pitfalls of our surgical technique stem from strict inclusion criteria for eligible patients. Cases with large tumors spreading into the lacrimal system and paranasal sinuses may be more suitable for conventional exenteration.

SCC is the most common nonpigmented malignancy of the ocular surface, which primarily occurs as a localized lesion confined to the epithelial layer and finally progresses to an invasive phenotype that breaks through the basement membrane.^[11]^ The incidence of SCC is about 9 to 10 times higher in the African population than in the Caucasian population.^[12]^ A retrospective cross-sectional study demonstrated that advanced stage of SCC was an independent variable of disease-related death.^[13]^ CM is the most common pigmented malignancy, which develops from precursor lesions such as primary acquired melanosis with atypia (75%) and nevus (20%) or de novo (5%).^[14]^ The age-adjusted incidence of CM is 0.49 per million in non-Hispanic whites, 0.33 in Hispanics, 0.18 in blacks, 0.17 in American Indians, and 0.15 in Asians.^[15]^ These life-threatening conditions are potentially surgical curable with orbital exenteration. During the past decades, the surgical principle dramatically progressed, shifting from radically extensive resection to a less invasive approaches.^[16]^ Shields reviewed the medical records of patients who underwent orbital exenteration, reaching a conclusion that eyelid-sparing technique can be used in most of malignant tumor cases to achieve rapid healing while still maintains the local tumor control rate.^[6]^ This technique preserves the eyelid skin but removes most of the orbital contents including the globe, making it difficult to reconstruct eye socket and to retain eyelid function. The modified approach reported in this study not only preserves the eyelid skin but also spares most of the orbital contents, enabling secondary reconstruction of the eye socket to obtain satisfied eyelid function, successful prothesis fitting and improved cosmetic results. There is growing evidence supports the concept that the surgical plan of exenteration should be tailored in each case so as to achieve rapid rehabilitation and satisfying esthetic outcome in patients with advanced ocular surface malignancies.

Tissue-preserving technique not only minimizes the deforming effect but also preserves the curable chance for ocular surface malignancies. In previous studies, the cumulative mortality rate of advanced CM was reported as 31.6% at 5 years of follow-up.^[17]^ The overall survival rate after conventional orbital exenteration varied from to 45% to 65% in patients with ocular surface malignancies.^[6,16]^ The follow-up data in our study provided evidence that individualized treatment exerts no negative effects on curable chance. The key point to secure survival rate is to completely remove tumor with no-touch technique. According to our protocol, in order to avoid iatrogenic dissemination of tumor cells, the upper and lower eyelids were sutured together immediately after skin incision. This maneuver, however, blocks direct observation of the tumor, making it difficult to clarify the deep margin. To solve this problem, a staining reagent named methylene blue was adapted to mark the epithelial layer of conjunctival sac. Similar dying techniques have been utilized in many other conditions. For instance, our team applied methylene blue staining to assist surgical removal of complex orbital dermoid cysts and has achieved favorable outcome.^[18]^ Many other reagents such as indocyanine green and trypan blue are available for orbital surgery.^[19]^ Further study is necessary to explore the penetrance and persistence of different dying reagents in surgical operations.

## 5 Conclusion

Our study reported a modified approach to orbital exenteration, which enables eye socket reconstruction to improve aesthetic results while still maintains the tumor control effects. This innovative technique is efficient and effective, and the surgical procedures are standardized and simplified for skilled practitioners. Advanced ocular surface tumors with no invasion of lacrimal system are eligible for this operation. More data are anticipated via longer follow-up duration and larger sample size to further validate the safety and efficacy of this novel technique.

## Author contributions

**Data curation:** Ruiqi Ma.

**Formal analysis:** Ruiqi Ma, Lu Gan.

**Investigation:** Lu Gan.

**Methodology:** Lu Gan.

**Resources:** Jie Guo.

**Supervision:** Xiaofeng Li, Jiang Qian.

**Validation:** Xiaofeng Li.

**Writing – original draft:** Ruiqi Ma, Jie Guo.

**Writing – review & editing:** Ruiqi Ma, Jie Guo, Jiang Qian.

## Supplementary Material

Supplemental Digital Content

## Supplementary Material

Supplemental Digital Content

## References

[1] Dai XZ Wang LY Shan Y. Clinicopathological analysis of 719 pediatric and adolescents’ ocular tumors and tumor-like lesions: a retrospective study from 2000 to 2018 in China. Int J Ophthalmol 2020;13:1961–7. 33344197 10.18240/ijo.2020.12.18 PMC7708371

[2] Shields CL Shields JA. Tumors of the conjunctiva and cornea. Indian J Ophthalmol 2019;67:1930–48. 31755426 10.4103/ijo.IJO_2040_19 PMC6896532

[3] Yin VT Merritt HA Sniegowski M. Eyelid and ocular surface carcinoma: diagnosis and management. Clin Dermatol 2015;33:159–69. 25704936 10.1016/j.clindermatol.2014.10.008

[4] Jayaprakasam A Vahdani K Rose GE. Rapid rehabilitation with skin–muscle sparing orbital exenteration: a single-center series. Ophthalmic Plast Reconstr Surg 2021;37:51–4. 32379171 10.1097/IOP.0000000000001677

[5] Baum SH Oeverhaus M Saxe F. Modified types of orbital exenteration, survival, and reconstruction. Graefes Arch Clin Exp Ophthalmol 2020;258:2305–12. 32572608 10.1007/s00417-020-04812-7

[6] Shields JA Shields CL Demirci H. Experience with eyelid-sparing orbital exenteration: the 2000 Tullos O. Coston lecture. Ophthalmic Plast Reconstr Surg 2001;17:355–61. 11642492 10.1097/00002341-200109000-00010

[7] Ben Simon GJ Schwarcz RM Douglas R. Orbital exenteration: one size does not fit all. Am J Ophthalmol 2005;139:11–7. 15652823 10.1016/j.ajo.2004.07.041

[8] Ma R Ren H Zhou X. Orbital exenteration for conjunctival melanoma: comparison of long-term outcome between individualised and conventional techniques. Eye (Lond) 2021;35:3410–8. 33608640 10.1038/s41433-021-01454-9 PMC8602404

[9] Bhattacharjee K Bhattacharjee H Kuri G. Comparative analysis of use of porous orbital implant with mucus membrane graft and dermis fat graft as a primary procedure in reconstruction of severely contracted socket. Indian J Ophthalmol 2014;62:145–53. 24618485 10.4103/0301-4738.128593 PMC4005228

[10] Wei YH Liao SL. The reconstruction of a contracted eye socket using a post-auricular full-thickness skin graft. Graefes Arch Clin Exp Ophthalmol 2014;252:821–7. 24599661 10.1007/s00417-014-2600-z

[11] Cicinelli MV Marchese A Bandello F. Clinical management of ocular surface squamous neoplasia: a review of the current evidence. Ophthalmol Ther 2018;7:247–62. 30030703 10.1007/s40123-018-0140-z PMC6258579

[12] Röck T Bartz-Schmidt KU Bramkamp M. Clinical management of squamous cell carcinoma of the conjunctiva. Am J Case Rep 2020;21:e919751. 32005796 10.12659/AJCR.919751 PMC7011170

[13] El-Hadad C Rubin ML Nagarajan P. Prognostic factors for orbital exenteration, local recurrence, metastasis, and death from disease in conjunctival squamous cell carcinoma. Ophthalmic Plast Reconstr Surg 2021;37:262–8. 33009325 10.1097/IOP.0000000000001798 PMC7865015

[14] Gallo B Thaung C Hay G. Invasive conjunctival melanoma mimicking ocular surface squamous neoplasia: a case series. Br J Ophthalmol 2021;105:775–8. 32675060 10.1136/bjophthalmol-2019-315393

[15] Grimes JM Shah NV Samie FH. Conjunctival melanoma: current treatments and future options. Am J Clin Dermatol 2020;21:371–81. 31965542 10.1007/s40257-019-00500-3

[16] Kiratli H Koç İ. Orbital exenteration: Institutional review of evolving trends in indications and rehabilitation techniques. Orbit 2018;37:179–86. 29039986 10.1080/01676830.2017.1383466

[17] Jain P Finger PT Damato B. Multicenter, international assessment of the eighth edition of the American Joint Committee on cancer cancer staging manual for conjunctival melanoma. JAMA Ophthalmol 2019;137:905–11. 31169891 10.1001/jamaophthalmol.2019.1640 PMC6555476

[18] Ma R Qian J Gan L. A modified methylene blue staining technique assists in the removal of complex orbital dermoid cysts. Chin J Optom Ophthalmol Vis Sci 2019;21:308–13.

[19] Boyle NS Chang EL. Intraoperative use of indocyanine green and trypan blue mixed with fibrin glue in the excision of periocular cystic lesions. Am J Ophthalmol Case Rep 2020;20:100990. 33251377 10.1016/j.ajoc.2020.100990 PMC7683233

